# Comorbid anxiety, loneliness, and chronic pain as predictors of intervention outcomes for subclinical depressive symptoms in older adults: evidence from a large community-based study in Hong Kong

**DOI:** 10.1186/s12888-024-06281-2

**Published:** 2024-11-21

**Authors:** Stephanie Ming Yin Wong, Dara Kiu Yi Leung, Tianyin Liu, Zuna Loong Yee Ng, Gloria Hoi Yan Wong, Wai Chi Chan, Terry Yat Sing Lum

**Affiliations:** 1 Department of Social Work and Social Administration, The University of Hong Kong, Hong Kong, China; 2 Department of Applied Social Sciences, The Hong Kong Polytechnic University, Hong Kong, China; 3 School of Psychology and Clinical Language Sciences, University of Reading, Reading, UK; 4 Department of Psychiatry, School of Clinical Medicine, LKS Faculty of Medicine, The University of Hong Kong, Hong Kong, China

**Keywords:** Depressive symptoms, Intervention outcome, Stepped-care intervention, Older adults, Anxiety, Loneliness

## Abstract

**Background:**

Depression is among the leading causes of the global burden of disease and is associated with substantial morbidity in old age. The importance of providing timely intervention, particularly those with subclinical symptoms, has thus increasingly been emphasised. Despite their overall effectiveness, a small but notable subgroup tends to be less responsive to interventions. Identifying predictors of non-remission and non-response is critical to inform future strategies for optimising intervention outcomes.

**Methods:**

A total of 4153 older adults aged 60 years and above with subclinical depressive symptoms (Patient Health Questionnaire-9 [PHQ-9] = 5–19) were recruited from JC JoyAge, a large-scale collaborative stepped-care intervention service across Hong Kong. A wide range of clinical and modifiable risk and protective factors at baseline were assessed, including depressive symptoms, anxiety symptoms, loneliness, suicidal ideation, cognitive capacity, multimorbidity, chronic pain, need for informal care due to mental health reasons, history of abuse, and sociodemographic characteristics. Separate multivariable logistic regression models were applied to identify predictors of non-remission (PHQ-9 ≥ 5) and non-response (< 50% reduction in PHQ-9) following intervention.

**Results:**

The rates of non-remission and non-response were 18.9% (*n* = 784) and 23.0% (*n* = 956), respectively. Comorbid anxiety symptoms (adjusted odds ratio [aOR] = 2.08, CI = 1.72–2.51; 1.28, 1.05–1.57), loneliness (2.00, 1.66–2.42; 1.67, 1.38–2.01), need for informal care (1.86, 1.49–2.33; 1.48, 1.18–1.85), lower cognitive capacity (0.95, 0.93–0.97; 0.94, 0.92–0.96), and absence of chronic pain (0.59, 0.48–0.72; 0.76, 0.64–0.91) predicted both non-remission and non-response. Meanwhile, moderate-to-severe depressive symptoms predicted higher odds of non-remission (1.41, 1.18–1.69) and lower odds of non-response (0.28, 0.23–0.34), respectively. Subgroup analyses conducted separately in older adults with mild and moderate-to-severe depressive symptoms at baseline revealed that comorbid anxiety, loneliness, need for informal care, and absence of chronic pain were consistent predictors of non-remission. Those with non-remission and non-response showed more depression-related functional impairments and poorer health-related quality of life post-intervention.

**Conclusions:**

Older adults with subclinical depressive symptoms showing comorbid anxiety, higher loneliness, need for informal care, and chronic pain may be offered more targeted interventions in future services. A personalised risk-stratification approach may be helpful.

**Trial registration:**

ClinicalTrials.gov identifiers: NCT03593889 (registered 29 May 2018), NCT04863300 (registered 23 April 2021).

**Supplementary Information:**

The online version contains supplementary material available at 10.1186/s12888-024-06281-2.

## Background

Depression is one of the leading causes of the global burden of disease [1] and a major risk factor for suicide deaths [2–4]. Aside from its long-term impact on the functioning and quality of life of affected individuals and their families and peers [5, 6], the longitudinal course of depression also inflicts considerable burdens on the healthcare system and economy, particularly if untreated or not fully treated [7–9]. The importance of timely intervention targeting those with subclinical depressive symptoms in the community is critical [10].

Despite a growing body of work in support of the effectiveness of community-based interventions in reducing depressive symptoms versus care-as-usual, studies have shown that around half of the participants fail to achieve remission at the end of service [11–14]. The engagement of at-risk populations in mental health services remains a persistent challenge due to stigma, service costs, and accessibility issues [15], and the inability to respond optimally to such interventions could further increase reluctance in service continuation. Further, non-response to early interventions can also indicate service mismatch and may increase the complexity of the condition and the risk of treatment resistance. Yet, little is known about the predictors of non-remission and non-response in subclinical populations. Such an exploration is critical for informing the design of more targeted and personalised strategies to optimise the effectiveness and cost-effectiveness of early intervention for at-risk populations in the community; the present study aims to fill this gap.

In contrast to the scarcity of research on clinical responsiveness to community-based interventions among non-clinical populations, treatment-resistant depression has been extensively studied owing to its substantial associated clinical, economic, and societal burden [16–19]. Generally defined as the inability to adequately respond to two or more trials of antidepressant pharmacotherapy, prior work has suggested that treatment resistance affects up to 30% of patients with major depressive disorder [20]. While identifying neurobiological markers of medication non-response had been a major focus in the previous literature on treatment-resistant depression, there is now consensus that interactions between multiple biological, psychosocial, and cultural factors contribute to this outcome [21, 22]. This increasing emphasis on a biopsychosocial approach to understanding suboptimal treatment response offers a practical framework for informing plausible prognosis, clinical management, and treatment options [23].

Notably, the comorbid presence of other mental disorders and risk factors has been suggested to influence both the aetiology and prognosis of depression, which raises the question of how treatment could be modified to best facilitate response and long-term remission, particularly when most treatments tend to focus on single mental disorders. To date, clinical factors including greater severity of current depressive symptoms, longer duration and recurrence of depressive episodes, comorbid anxiety disorder, physical illnesses, suicidality, as well as socioenvironmental factors such as major negative life events (e.g., maltreatment history), have been found to be more consistent predictors of treatment-resistant depression [24–27]. Several studies have identified cognitive impairment to be associated with greater risks of poorer treatment response [28], including in outpatients with moderate-to-severe depression [29], which may be explained by reduced cognitive flexibility [30] and learning capacity [31].

Several recent studies have expanded this line of investigation from patients in clinical settings to those in the community and primary or collaborative care settings [11, 32–35]. These studies similarly highlighted the roles of comorbid anxiety, loneliness, multiple chronic medical conditions, and lower socioeconomic status in predicting non-remission in these samples [11, 32–35]. While they have not explicitly conceptualised the possible mechanisms underlying their influences on intervention outcomes, several factors might play important roles. For instance, generalised anxiety disorder and depression are known to be forms of internalising disorders that show high degrees of shared genetic risk and common sets of risk factors [36]. Although psychological interventions (e.g., cognitive-behaviour therapy [CBT]) are generally effective in reducing both depressive and anxiety symptoms [37], the presence of elevated anxiety symptoms pre-intervention may indicate greater levels of emotional avoidance and dysregulation, interpersonal problems, intolerance of uncertainty, and persistent worries, which might require the incorporation of more targeted approaches beyond regular interventions for depression into the care plan [38]. Similarly, the influence of loneliness on hypervigilance to social stress and elevated stress sensitivity, as well as negative cognitive schemas, also play important roles in the maintenance of depressive symptoms, hence poorer intervention outcomes [39].

Interestingly, the effects of initial depressive symptom severity and recurrent depressive episodes on intervention outcomes in community samples have been less consistent [11, 14, 40, 41]. A study exploring predictors of the time to clinical remission and response (defined as Patient Health Questionnaire [PHQ-9] < 5 and ≥ 50% reduction in PHQ-9 scores, respectively) among a sample of older adults in a home/community-based depression care management programme found that more severe symptoms of depression were predictive of a lower odds of remission (i.e., non-remission), with no significant effect observed on clinical response [14]. Notably, this study found that higher educational attainment and income level were among the only factors associated with both outcomes, although only a limited range of non-modifiable demographic variables and depressive symptom severity were accounted for [14]. The early identification of modifiable factors in predicting the likelihood of achieving remission would be crucial for informing the design of more optimal interventions to improve individual outcome trajectories [42].

Building on evidence from previous work, we aimed to identify potentially modifiable predictors of clinical non-remission and non-response in a large sample of older adults with subclinical depressive symptoms from a community-based collaborative stepped-care intervention in Hong Kong. We hypothesised that anxiety and loneliness, given their high degree of overlapping genetics and cognitive mechanisms with depression, would show the strongest association with poorer intervention response, as defined by higher odds of non-remission and non-response. With further reference from the literature on treatment-resistant depression, we anticipated that greater cognitive impairments, multimorbidity and chronic pain, need for informal care due to mental health reasons, and suicidal ideation would also be predictive of non-remission and non-response. Meanwhile, higher depressive symptoms were anticipated to be predictive of higher odds of non-remission and lower odds of non-response. We also anticipated that those who failed to achieve remission and response would show more functional impairments related to depressive symptoms and poorer health-related quality of life (HR-QoL).

## Methods

### Participants and study design

This study included 4153 participants recruited from the Jockey Club Holistic Support Project for Elderly Mental Wellness (JC JoyAge), which is to date the first initiative in Hong Kong to adopt a community-based collaborative stepped-care approach to early intervention for older adults with mild-to-moderate levels of depressive symptoms (median service duration = 9 months, IQR = 7–12 months). Details of the JoyAge model have been reported [8, 43, 44]. The implementation of JoyAge has expanded from four districts in 2016–2019 (Phase I) to all 18 districts in 2020–2023 (Phase II), involving 47 district-based elderly community centres and community-based mental wellness centres. All local residents aged 60 years and above, with mild-to-moderate levels of depressive symptoms (PHQ-9 score of 5–19), and with no known history of schizophrenia spectrum disorder, bipolar disorder, autism spectrum disorder, intellectual disability, Parkinson’s disease, or major neurocognitive disorders were eligible. Those with significant suicidal risk (determined using items concerning any present suicidal thoughts, plan, or attempt, respectively, item nine of the PHQ-9 concerning self-harm and suicidal thoughts, as well as via clinical assessment) were excluded and referred to local hospital psychiatric services or provided with additional support following standard risk management protocols.

Following the stepped-care approach, participants were provided with relevant psychotherapeutic interventions according to their depressive symptom severity, with CBT being the main intervention modality. Those with mild symptoms (PHQ-9 = 5–9) were offered low-intensity group-based CBT (6 sessions), while those with moderate-to-moderately severe symptoms (PHQ-9 = 10–19) were offered high-intensity group-based CBT (8 sessions) or individual-based interventions when assessed to be required. More details of the CBT protocol in the JoyAge project are provided in Supplementary Material S1. To ensure the model can be more widely adopted and is scalable in the community, all interventions were provided by trained social workers, with ongoing supervision by the JoyAge clinical team (involving clinical psychologists and senior social workers, with psychiatric consultants). After the first intervention trial, participants may be stepped up or down based on their post-intervention symptom severity. Those who showed no major symptoms (PHQ-9 < 5), no significant symptoms of anhedonia and depressed mood (PHQ-9 items 1 and 2 < 2), achieved one or more personal recovery goals, showed no major loneliness symptoms (3-item UCLA Loneliness Scale [UCLA3] < 3), and showed improvements in protective factors (e.g., increased connections with community-based social services, enlarged social circle) were considered to have met the criteria for “discharge” from the programme. A service duration of no more than 9 months is recommended to ensure the service is time-limited and can be more widely scaled up. Participants who were unable to reach clinical remission in this study were those who received at least two trials of psychological intervention in JoyAge but continued to show mild or above levels of depressive symptoms, similar to previous definitions of treatment-resistant depression (PHQ-9 ≥ 5) [11].

During Phase I of the project, older adults with no major depressive symptoms (PHQ-9 < 5) but with major risk factors (e.g., high levels of loneliness, lack of social interaction and meaningful activities, recent bereavement) were also eligible. Given the growing population of older adults at risk of depression, the project has shifted its focus to those with at least mild-to-moderately severe symptoms of depression (PHQ-9 = 5–19), although the clinical service model and protocol remained unchanged and were regularly monitored by the same JoyAge clinical team. In the present study, we analysed data from those who met the criteria for PHQ ≥ 5 to align with the updated clinical service protocol in Phase II. Findings concerning the effectiveness of the JoyAge intervention will be reported elsewhere (ClinicalTrials.gov Identifiers: NCT03593889, NCT04863300; registered May 2018 and April 2021, respectively). Informed consent was obtained from all participants, with ethics approval granted by the Human Research Ethics Committee (HREC) of The University of Hong Kong (Phase I reference number: EA1709021; Phase II reference number: EA2003001). All procedures contributing to this work complied with the Helsinki Declaration of 1975, as revised in 2000.

### Measures

Depressive symptoms during the previous two weeks were assessed using the PHQ-9 [45], which comprises nine items based on the criteria for major depression in the DSM-IV and is also recommended in the DSM-V. All items are rated on a 4-point Likert scale (from 0 “not at all” to 3 “nearly every day”), with a cut-off score of ≥ 10 indicating moderate-to-severe symptoms [45]. The Chinese version of the PHQ-9 has been validated in Hong Kong [46]. The Cronbach’a alpha (α) of the scale was 0.58 and 0.66 at baseline and post-intervention, respectively. Following prior work, clinical non-remission was defined as a score of PHQ-9 ≥ 5 post-intervention, while non-response was defined as < 50% reduction in depressive symptoms from the time of initial assessment to post-intervention [11, 14, 47, 48].

Anxiety symptoms during the previous two weeks were assessed using the Generalized Anxiety Disorder Scale (GAD-7) [49], which comprises seven items based on the DSM-IV criteria that also correspond to the DSM-V criteria. Items are rated on a 4-point Likert scale (from 0 “not at all” to 3 “nearly every day”), with a cut-off score of ≥ 10 indicating moderate-to-severe symptoms [49]. The Chinese version of the GAD-7 has been widely adopted, including in older adults [50, 51]. The internal consistency of the scale was good in this study (α = 0.89).

Loneliness symptoms were assessed using the 3-item UCLA Loneliness Scale (UCLA-3) [19], which is one of the most widely adopted measures of loneliness [52] and has been validated in Chinese older adults [53] (α = 0.87 in this study). The three main symptoms of feelings of “lacking companionship”, “left out”, and “isolated” were captured and were rated on a 4-point Likert scale (from 0 “never” to 3 “often”). To facilitate early risk detection in routine care settings, we dichotomised scores into those with low (< 7) and high levels of loneliness (≥ 7), as in previous studies investigating the impact of loneliness [54–56].

Cognitive capacity was assessed using the Montreal Cognitive Assessment 5-min protocol (MoCa 5-min), which provides a composite index of cognition based on five main domains: attention, verbal learning and memory, executive functions/language, and orientation [57]. The MoCa 5-min has been validated in Hong Kong and has been shown to have good reliability [57] (α = 0.66 in this study).

Multimorbidity, defined as four or more chronic diseases, was assessed using a binary yes/no item in Phase I of JoyAge. In Phase II, a more extensive checklist of 12 chronic diseases was used: chronic lung disease (e.g., chronic obstructive pulmonary disease), kidney disease/nephropathy, heart disease, angina pectoris, congestive heart failure, cancer, hypertension, diabetes, arthritis, asthma, stroke, and others. To align with data from Phase I, the presence of four or more of these diseases was dichotomised into a yes/no outcome. Chronic pain was assessed using a yes/no item in both phases.

The need for informal care for mental health reasons during the previous three months – as a reflection of greater mental health-related impairments in daily functioning – was assessed using an item from the locally adapted Client Service Receipt Inventory (CSRI) [8, 58]. Participants were asked the average hours per week they required help from their friends or relatives to undertake tasks related to personal care (e.g., washing, dressing), household chores (e.g., cooking, cleaning), tasks outside the home (e.g., shopping, transport), or other tasks because of emotional problems. A response of one hour or more was defined as requiring need for informal care.

Aside from sex, age, and years of education, participants were also asked about their residential status (living alone/with others) and whether they were receiving Comprehensive Social Security Assistance (CSSA) – one of the major social security schemes in Hong Kong – as a reflection of their socioeconomic status (SES) similar to previous work [15]. As additional indicators of vulnerability, any diagnosis of depressive or anxiety disorder, history of abuse, and current suicidal ideation were also assessed using a checklist.

An item was included after the PHQ-9 to assess the degree to which the depressive symptoms experienced caused difficulties in everyday life functioning (including occupational, daily, or social functioning), rated on a 4-point Likert scale (from 0 “not difficult at all” to 3 “extremely difficult”) [45, 59]. In addition, health-related quality of life (HR-QoL) was assessed using the EuroQol-5D (EQ-5D-5D) [60, 61], the internal consistency of which was also found to be good (α = 0.77).

### Statistical analysis

Descriptive statistics for all variables were first generated in the whole sample and among those who did and did not show clinical remission and response post-intervention, respectively. Then, we examined changes in depressive symptom severity and the proportions with mild, moderate, and moderate-to-severe depressive symptoms from baseline to post-intervention using related-samples Wilcoxon signed rank test and McNemar test. Chi-square tests or Mann–Whitney U Tests were applied to examine univariate prospective associations among the range of baseline risk and protective factors and both non-remission and non-response. Two separate multivariable logistic regression models were then applied to examine the independent contributions of all plausible predictors to non-remission and non-response. Further, we separately applied these multivariable models among subgroups of those with mild symptoms (PHQ-9 = 5–9) and moderate-to-severe symptoms (PHQ-9 = 10–19) at baseline, respectively, to examine whether the patterns of associations would differ as a function of initial depressive symptom severity. Lastly, the associations of non-remission and non-response with depression-related functioning and HR-QoL were tested through a series of Mann–Whitney U tests. Effect sizes were reflected by adjusted odds ratio (aOR) with their 95% confidence intervals (CIs) in the logistic regression models. SPSS version 29.0 was used for all analyses.

## Results

### Sample characteristics

**Fig. 1:**
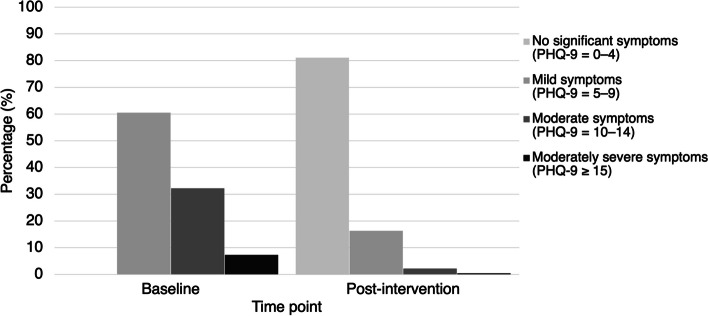
Changes in rates of depressive symptoms following the collaborative stepped-care intervention (*n* = 4153). *Note*. PHQ-9 = Patient Health Questionnaire

Table 1 shows the sample characteristics. The mean sample age was 75.3 years (SD = 8.0), with the majority (82.7%) being females. The depressive symptom severity of the sample showed substantial reductions, from 9.03 (SD = 3.44) at baseline to 3.20 (SD = 2.50) post-intervention, *p* < 0.001. While all participants scored 5 or above on the PHQ-9 at baseline, the majority no longer scored above this cut-off post-intervention, with 18.9% continuing to meet this criterion at the time of intervention completion (i.e., non-remission) (Fig. 1). The proportion of participants with moderate-to-severe depressive symptoms (PHQ-9 ≥ 10) also significantly reduced from 39.5% (*n* = 1640) to 2.6% (*n* = 107), *p* < 0.001. 23.0% (*n* = 956) were considered to have shown non-response (< 50% reduction in PHQ-9 scores).

**Table 1:** Baseline sociodemographic, psychosocial, and clinical characteristics of the sample

	Whole Sample (*n* = 4153)	Clinical remission^a^			Clinical response^b^		
		Yes (*n* = 3369)	No (*n* = 784)	*p*	Yes (*n* = 3197)	No (*n* = 956)	*p*
**Sociodemographics**							
Female sex, n (%)	3434 (82.7)	2792 (82.9)	642 (81.9)	0.51	2644 (82.7)	790 (82.6)	0.96
Age	75.3 (8.0)	75.2 (7.9)	75.8 (8.4)	0.052	**75.0 (7.9)**	**76.5 (8.1)**	**< .001**
Years of education	5.9 (4.6)	**5.95 (4.52)**	**5.56 (4.77)**	**0.007**	**5.97 (4.45)**	**5.55 (4.95)**	**< .001**
Living alone, n (%)	1623 (39.1)	1311 (38.9)	312 (39.8)	0.65	1224 (38.3)	399 (41.7)	0.055
Receiving social security assistance, n (%)	1008 (24.3)	**766 (22.7)**	**242 (30.9)**	**< 0.001**	**731 (22.9)**	**277 (29.0)**	**< 0.001**
**Prior stress exposure**							
History of abuse, n (%)	157 (3.8)	**108 (3.2)**	**49 (6.3)**	**< 0.001**	**122 (3.8)**	**35 (3.7)**	**< 0.001**
**Need for informal care, n (%)**							
Informal care required for mental health reasons	530 (12.8)	**372 (11.0)**	**158 (20.2)**	**< 0.001**	**368 (11.5)**	**162 (16.9)**	**< 0.001**
**Psychiatric and physical illness history, n (%)**							
Has a diagnosis of depression/anxiety	719 (17.3)	571 (16.9)	148 (18.9)	0.20	604 (18.9)	115 (12.0)	0.20
Has multimorbidity (≥ 4 chronic diseases)	268 (6.5)	229 (6.8)	39 (5.0)	0.061	219 (6.9)	49 (5.1)	0.057
Has chronic pain	1243 (29.9)	**1066 (31.6)**	**177 (22.6)**	**< .001**	**999 (31.2)**	**244 (25.5)**	**< .001**
**Cognition**							
Overall cognitive ability (MoCA 5-min)	22.40 (4.41)	**22.62 (4.30)**	**21.42 (4.75)**	**< 0.001**	**22.69 (4.29)**	**21.42 (4.68)**	**< 0.001**
**Suicidal ideation, n (%)**							
Current suicidal ideation	728 (17.5)	**512 (15.2)**	**216 (27.6)**	**< 0.001**	573 (17.9)	155 (16.2)	0.22
**Mental health symptoms, n (%)**							
Moderate-to-severe depression (PHQ-9 ≥ 10)	1640 (39.5)	**1228 (36.4)**	**412 (52.6)**	**< 0.001**	**1438 (45.0)**	**202 (21.1)**	**< 0.001**
Moderate-to-severe anxiety (GAD-7 ≥ 10)	1001 (24.1)	**680 (20.2)**	**321 (40.9)**	**< 0.001**	788 (24.6)	213 (22.3)	0.13
High loneliness (UCLA3 ≥ 7)	859 (20.7)	**585 (17.4)**	**274 (34.9)**	**< 0.001**	**608 (19.0)**	**251 (26.3)**	**< 0.001**

Statistics significant at the *p* < 0.05 level are boldfaced. *GAD-7 *7-item Generalized Anxiety Disorder scale, *MoCA 5-min *Montreal Cognitive Assessment 5-min, *PHQ-9 *9-item Patient Health Questionnaire, *UCLA3 *UCLA 3-item Loneliness Scale

^a^Clinical remission is defined as PHQ-9 < 5 post-intervention

^b^Clinical response is defined as remission is defined as ≥ 50% reduction in PHQ-9 post-intervention

### Factors associated with non-remission and non-response in the whole sample

Table 1 presents findings from the univariate analyses, while Table 2 presents findings from the multivariable models showing factors associated with non-remission and non-response in the whole sample. Accounting for all other variables, high loneliness (aOR = 2.00, CI = 1.66–2.42; aOR = 1.67, CI = 1.38–2.01), moderate-to-severe anxiety symptoms (2.08, 1.72–2.51; 1.28, 1.05–1.57), and need for informal care for mental health reasons (1.86, 1.49–2.33; 1.48, 1.19–1.85) at baseline predicted both non-remission and non-response, respectively (Table 2). Meanwhile, higher cognitive capacity (aOR = 0.95, CI = 0.93–0.97; aOR = 0.94, 0.92–0.96) and chronic pain (0.59, 0.48–0.72; 0.76, 0.64–0.91) predicted lower odds of both outcomes. Receiving social security assistance (aOR = 1.35, CI = 1.11–1.65), a history of abuse (1.49, 1.02–2.18), and suicidal ideation (1.32, 1.08–1.62) specifically predicted associated with non-remission, while multimorbidity (aOR = 0.70, CI = 0.50–0.98) and a prior diagnosis of depression or anxiety (0.78, 0.62–0.98) predicted lower odds of non-response (Table 2). Whereas higher depressive symptoms at baseline were associated with higher odds of non-remission (aOR = 1.41, CI = 1.18–1.69), they were associated with lower odds of non-response (0.28, 0.23–0.34).

**Table 2:** Multivariable logistic regression showing factors associated with non-remission and non-response

	Whole sample (*n* = 4153)					
	Non-remission^a^ (*n* = 784)			Non-response^b^ (*n* = 956)		
	aOR	95% CI	*p*	aOR	95% CI	*p*
**Personal background factors**						
Male sex	(Ref)			(Ref)		
Female sex	1.09	0.87–1.35	0.47	1.15	0.93–1.41	0.19
Age	1.00	0.99–1.02	0.67	1.01	0.99–1.02	0.42
Years of education	1.01	0.99–1.03	0.32	1.01	0.99–1.03	0.19
Not receiving social security assistance	(Ref)			(Ref)		
Receiving social security assistance	**1.35**	**1.11–1.65**	**0.002**	1.19	0.99–1.43	0.061
Living with others	(Ref)			(Ref)		
Living alone	0.85	0.71–1.01	0.070	0.97	0.82–1.14	0.70
**Prior stress exposure**						
No history of abuse	(Ref)			(Ref)		
Has history of abuse	**1.49**	**1.02–2.18**	**0.037**	1.32	0.88–1.97	0.19
**Psychiatric and physical illness history**						
No diagnosis of depression/anxiety	(Ref)			(Ref)		
Has diagnosis of depression/anxiety	0.87	0.69–1.08	0.21	**0.78**	**0.62–0.98**	**0.035**
No multimorbidity (< 4 chronic diseases)	(Ref)			(Ref)		
Has multimorbidity (≥ 4 chronic diseases)	0.85	0.58–1.23	0.39	**0.70**	**0.50–0.98**	**0.037**
No chronic pain	(Ref)			(Ref)		
Has chronic pain	**0.59**	**0.48–0.72**	**< 0.001**	**0.76**	**0.64–0.91**	**0.002**
**Need for informal care**						
No informal care required for mental health reasons	(Ref)			(Ref)		
Informal care required for mental health reasons	**1.86**	**1.49–2.33**	**< 0.001**	**1.48**	**1.19–1.85**	**< 0.001**
**Cognition**						
Cognitive capacity (MoCA 5-min)	**0.95**	**0.93–0.97**	**< 0.001**	**0.94**	**0.92–0.96**	**< 0.001**
**Current suicidal ideation**						
No suicidal ideation	(Ref)			(Ref)		
Has suicidal ideation	**1.32**	**1.08–1.62**	**0.007**	1.04	0.84–1.29	0.72
**Mental health symptoms**						
No-to-mild depressive symptoms (PHQ-9 < 10)	(Ref)			(Ref)		
Moderate-to-severe depressive symptoms (PHQ-9 ≥ 10)	**1.41**	**1.18–1.69**	**< 0.001**	**0.28**	**0.23–0.34**	**< 0.001**
No-to-mild anxiety symptoms (GAD-7 < 10)	(Ref)			(Ref)		
Moderate-to-severe anxiety symptoms (GAD-7 ≥ 10)	**2.08**	**1.72–2.51**	**< 0.001**	**1.28**	**1.05–1.57**	**0.015**
Low loneliness (UCLA3 < 7)	(Ref)			(Ref)		
High loneliness (UCLA3 ≥ 7)	**2.00**	**1.66–2.42**	**< 0.001**	**1.67**	**1.38–2.01**	**< 0.001**

Statistics significant at the *p* < 0.05 level are boldfaced. *GAD-7* 7-item Generalized Anxiety Disorder scale, *MoCA 5-min* Montreal Cognitive Assessment 5-min, *PHQ-9* 9-item Patient Health Questionnaire, *UCLA3* UCLA 3-item Loneliness Scale

^a^ Non-remission is defined PHQ-9 ≥ 5 post-intervention, which is indicative of failure to achieve clinical remission

^b^ Non-response is defined < 50% reduction in PHQ-9 post-intervention, which is indicative of failure to achieve clinical response

### Factors associated with non-remission and non-response among those with mild and moderate-to-severe depressive symptoms at baseline

Further analyses were conducted to examine whether the patterns of associations differed between those with different levels of depressive symptoms at baseline. Table 3 presents the detailed findings. In both subgroups, high anxiety (aOR range = 1.56–2.17) was among the only factors that consistently predicted both non-remission and non-response, while having chronic pain (aOR range = 0.60–0.81) predicted lower odds of both outcomes.

**Table 3:** Multivariable logistic regression showing factors associated with non-remission and non-response among those with mild symptoms and moderate-to-severe symptoms at baseline

	Mild depressive symptoms at baseline (PHQ-9 = 5–9) (*n* = 2513)						Moderate-to-severe depressive symptoms at baseline (PHQ-9 ≥ 10) (*n* = 1640)					
	Non-remission^a^ (*n* = 372)			Non-response^b^ (*n* = 754)			Non-remission^a^ (*n* = 412)			Non-response^b^ (*n* = 202)		
	aOR	95% CI	*p*	aOR	95% CI	*p*	aOR	95% CI	*p*	aOR	95% CI	*p*
**Personal background factors**												
Female sex	1.14	0.83–1.55	0.42	1.11	0.87–1.41	0.41	1.03	0.75–1.41	0.87	1.32	0.86–2.03	0.20
Age	1.00	0.98–1.02	0.98	1.00	0.99–1.02	0.69	1.00	0.99–1.02	0.61	1.01	0.99–1.03	0.43
Years of education	1.02	0.99–1.05	0.29	1.01	0.98–1.03	0.60	1.00	0.98–1.03	0.78	1.03	0.99–1.07	0.14
Receiving social security assistance	1.30	0.99–1.71	0.060	1.16	0.94–1.44	0.17	**1.38**	**1.04–1.84**	**0.026**	1.29	0.90–1.85	0.17
Living alone	0.86	0.66–1.11	0.25	0.94	0.78–1.14	0.55	0.83	0.64–1.08	0.16	1.04	0.74–1.44	0.83
**Prior stress exposure**												
Has history of abuse	0.77	0.32–1.86	0.56	0.67	0.34–1.32	0.25	**1.82**	**1.18–2.81**	**0.007**	**2.01**	**1.21–3.34**	**0.007**
**Psychiatric and physical illness history**												
Has diagnosis of depression/anxiety	**0.63**	**0.41–0.98**	**0.040**	**0.67**	**0.49–0.91**	**0.010**	0.97	0.74–1.28	0.84	0.91	0.64–1.30	0.60
Has multimorbidity (≥ 4 chronic diseases)	0.63	0.37–1.09	0.10	**0.59**	**0.40–0.87**	**0.007**	1.21	0.72–2.05	0.47	1.35	0.70–2.58	0.37
Has chronic pain	**0.60**	**0.45–0.80**	**< 0.001**	**0.81**	**0.66–1.00**	**0.044**	**0.60**	**0.46–0.79**	**< 0.001**	**0.67**	**0.47–0.95**	**0.026**
**Need for informal care**												
Informal care required due to mental health reasons	**2.29**	**1.68–3.11**	**< 0.001**	**1.48**	**1.14–1.92**	**0.003**	**1.40**	**1.01–1.96**	**0.046**	1.42	0.92–2.15	0.098
**Cognition**												
Cognitive capacity (MoCA 5-min)	**0.91**	**0.88–0.93**	**< 0.001**	**0.94**	**0.91–0.96**	**< 0.001**	1.00	0.97–1.03	0.87	0.96	0.92–1.00	0.051
**Suicidal ideation**												
Current suicidal ideation	**1.60**	**1.14–2.26**	**0.007**	1.00	0.74–1.34	0.98	1.29	1.00–1.67	0.051	1.13	0.81–1.57	0.48
**Mental health symptoms**												
Moderate-to-severe anxiety symptoms (GAD-7 ≥ 10)	**1.86**	**1.36–2.54**	**< 0.001**	1.06	0.81–1.39	0.65	**2.17**	**1.70–2.78**	**< 0.001**	**1.58**	**1.15–2.18**	**0.005**
High loneliness (UCLA3 ≥ 7)	**2.17**	**1.64–2.87**	**< 0.001**	**1.56**	**1.24–1.98**	**< 0.001**	**1.78**	**1.37–2.30**	**< 0.001**	**1.82**	**1.31–2.52**	**< 0.001**

Statistics significant at the *p* < 0.05 level are boldfaced. *GAD-7* 7-item Generalized Anxiety Disorder scale, *MoCA 5-min* Montreal Cognitive Assessment 5-min, *PHQ-9* 9-item Patient Health Questionnaire, *UCLA3* UCLA 3-item Loneliness Scale

^a^ Non-remission is defined PHQ-9 ≥ 5 post-intervention, which is indicative of failure to achieve clinical remission

^b^ Non-response is defined < 50% reduction in PHQ-9 post-intervention, which is indicative of failure to achieve clinical response

In the subgroup with mild depressive symptoms at baseline, requiring informal care for mental health reasons (aOR = 2.29, CI = 1.68–3.11; 1.48, 1.14–1.92) and lower cognitive capacity (0.91, 0.88–0.93; 0.94, 0.91–0.96) predicted higher odds of both non-remission and non-response, while a diagnosis of depression or anxiety predicted lower odds of the outcomes (0.63, 0.41–0.98; 0.67, 0.49–0.91, respectively). Furthermore, moderate-to-severe anxiety symptoms specifically predicted higher odds of non-remission (aOR = 1.86, CI = 1.36–2.54), while multimorbidity predicted lower odds of non-response (0.59, 0.40–0.87).

In the subgroup with moderate-to-severe depressive symptoms at baseline, moderate-to-severe anxiety symptoms (aOR = 2.17, CI = 1.70–2.78; 1.58, 1.15–2.18) and a history of abuse (1.82, 1.18–2.81; 2.01, 1.21–3.34) predicted higher odds of both non-remission and non-response. Meanwhile, the need for informal care (aOR = 1.40, CI = 1.01–1.96) and lower SES (1.38, 1.04–1.84) also significantly predicted non-remission but not non-response (Table 3).

### Non-remission and non-response and their associations with functioning and quality of life

In the whole sample, significant improvements were observed in levels of depression-related functional impairment (mean = 0.91 [SD = 0.58] to 0.43 [0.53] post-intervention) and HR-QoL (0.66 [0.25] to 0.75 [0.23]), both *p* < 0.001. Compared with those who achieved remission, those with non-remission showed higher levels of depression-related functional impairments (mean = 0.85 [0.54] *vs* 0.34 [0.48]) and poorer HR-QoL (0.58 [0.28] *vs* 0.79 [0.19]), both *p* < 0.001. Those who showed non-response also showed higher levels of functional impairment (mean = 0.74 [0.56] *vs* 0.34 [0.49]) and poorer HR-QoL (0.64 [0.27] *vs* 0.79 [0.20]) than those who showed clinical response, both *p* < 0.001.

## Discussion

Using data from a large sample of older adults from a territory-wide community-based collaborative stepped-care intervention for subclinical depressive symptoms, our study showed that comorbid anxiety symptoms, higher loneliness, need for informal care for mental health reasons, and lower cognitive capacity at baseline predicted non-remission and non-responsiveness to intervention. Notably, higher loneliness was among the only factors that remained significantly associated with poorer intervention outcomes both in those with mild and moderate-to-severe depressive symptoms at baseline, which suggests that its impact on outcomes is largely independent of initial depressive symptom severity. Meanwhile, a history of abuse served as a risk factor for elevated resistance to intervention particularly in those with moderate-to-severe depressive symptoms at baseline.

Our findings were generally consistent with previous work conducted on clinical samples [24, 26, 27]. As expected, the depressive symptoms of most participants considerably improved over the course of the intervention, although a small proportion was unable to achieve clinical remission or response. In line with a previous study conducted among older adults from a home/community-based collaborative care programme in the United States [14], we also found associations between lower SES and poorer intervention outcomes in our sample.

In line with our hypothesis, anxiety symptoms and loneliness at baseline showed relatively strong associations with suboptimal intervention outcomes. These observations are also consistent with previous studies conducted in both clinical and community-based samples [11, 27], which offers some support to the perspective that these phenomena share overlapping mechanisms with depression, and in turn, contribute to the maintenance of depressive symptoms. It would be important for future work to examine how interventions could best support people presenting both anxiety and depressive symptoms (e.g., first targeting anxiety followed by depression, first depression followed by anxiety, or designing an integrative intervention that targets both symptom dimensions). Future research may also examine whether placing greater emphasis on treating “bridge” or overlapping symptoms of depression and anxiety (such as sleep disturbances, poor concentration, and fatigue) [62] would improve intervention outcomes.

The finding that loneliness serves as a major contributor to poorer intervention outcomes is in line with the increasing efforts in research and clinical practice targeting the reduction of loneliness and social isolation [39, 63, 64], including the recent World Health Organization initiative on improving social connection as a global health priority [65] and the prioritisation of social interventions to address loneliness for difficult-to-treat depression as a national research priority in the UK [66]. There is clear evidence of the consequences of loneliness, social isolation, and disconnection on psychological and physical outcomes [67]. The benefits of adequate social support have also been reported [68]. Despite these, it is worth noting that loneliness can be experienced even among those with an identifiable social network (e.g., those who live with their family and actively participate in social activities [69], which is consistent with the cognitive perspective of loneliness as a response to perceived discrepancies between desired and actual levels of social connections [70, 71]. Given that loneliness is a modifiable factor, future research should consider exploring the effectiveness of loneliness-focused CBT for those with subclinical depression in optimising rates of clinical remission and response.

Interestingly, our findings suggested that those with chronic pain or multimorbidity showed more optimal intervention outcomes, which is in contrast to those reported in previous work [33]. According to the fear-avoidance model, catastrophising beliefs and fear related to pain can contribute to elevated pain intensity and emotional distress [72, 73]. It is possible that the CBT groups offered in JoyAge had targeted pain- or disease-related distress and their related negative cognitions in some older adults who reported chronic pain and multimorbidity as their chief complaints, which might have contributed to greater improvements in their depressive symptoms. The degree of response to intervention among those whose depressive symptoms were triggered by chronic disease or pain may also differ from those whose symptoms were triggered by other intrinsic or psychological factors. A further investigation into the mechanisms of change underlying CBT for older adults with both chronic health conditions and depressive symptoms, as compared to those with only depressive symptoms as their main presenting problems, is warranted.

As raised in previous studies, improving the precision in treatment selection for individual patients with depression is critical yet remains a major challenge in practice [74]. This study serves as an initiative to fill the literature gap by identifying early risk factors of poor response to community-based interventions among those with subclinical depressive symptoms. Aside from including initial depressive symptoms in the multivariable model, we also considered how the set of modifiable risk and protective factors may be differentially associated with intervention outcomes between those with mild or moderate-to-severe depressive symptoms at baseline to improve robustness of our observations. We further showed the burden of non-remission and response on the functioning and quality of life of older adults.

Despite these insights, we acknowledge several limitations. While recurrent depressive episodes and earlier onset have been identified as predictors of treatment resistance [24, 26, 27], such information was not available in the present study. Indeed, this can be more difficult to ascertain from older adults as their awareness of psychiatric problems is generally lower than in young adults [75, 76]. With greater mental health awareness among general public and advancements in public psychiatric services, such information might become more accessible (e.g., via electronic medical records for tracking psychiatric history from childhood) even among older adults in the future. While we identified a number of plausibly modifiable predictors of non-remission and non-response, further work is needed to elucidate the pathways underlying their interrelationships. The present sample also comprised a majority of female participants, similar to previous community-based interventions for depressive symptoms in older adults (e.g., [14]). Nevertheless, it would be important to examine whether a similar set of predictors of non-remission and non-response could be applied to male participants. The presentation of depressive symptoms and their aetiology in old age can also differ from those in younger age groups, which has not been fully explored. A further examination of the present study findings in other populations and regions is thus encouraged, which should inform more context-specific clinical decisions across populations. When possible, using data with a longer follow-up time frame would be helpful to further examine whether the set of predictors we identified could also inform the sustainability of intervention outcomes and relapse.

## Conclusion

Timely intervention for those with subclinical depressive symptoms is crucial; yet, maximising successful remission of symptoms is equally important. Adopting a more personalised approach to intervention by not only accounting for initial depressive symptoms but also the presence of other risk and protective factors may be important. Specifically, identifying older adults with comorbid anxiety symptoms, loneliness, and chronic pain and targeting these factors in future interventions may help optimise outcomes.

## Supplementary Information

Supplementary Material 1.

## Data Availability

De-identified data can be made available upon reasonable request and should be directed to the corresponding author.
