# Supplementary material for: Comorbid anxiety, loneliness, and chronic pain as predictors of intervention outcomes for subclinical depressive symptoms in older adults: evidence from a large community-based study in Hong Kong
Source: BMC Psychiatry. 2024 Nov 21;24:839. doi: 10.1186/s12888-024-06281-2 (PMC11580345; doi:10.1186/s12888-024-06281-2)
Supplement: Supplementary file 1 — Supplementary Material 1. [file 12888_2024_6281_MOESM1_ESM.pdf]

Supplementary Material

Comorbid anxiety, loneliness, and chronic pain as predictors of intervention outcomes for subclinical depressive symptoms in older adults: evidence from a large community-based study in Hong Kong

Stephanie Ming Yin Wong,[1] Dara Kiu Yi Leung,[1] Tianyin Liu,[2] Zuna Loong Yee Ng,[1] Gloria Hoi Yan Wong,[1,3] Wai Chi Chan,[4] Terry Yat Sing Lum[1]

[1] Department of Social Work and Social Administration, The University of Hong Kong, Hong Kong SAR, China
[2] Department of Applied Social Sciences, The Hong Kong Polytechnic University, Hong Kong SAR, China
[3] School of Psychology and Clinical Language Sciences, University of Reading, United Kingdom
[4] Department of Psychiatry, School of Clinical Medicine, LKS Faculty of Medicine, The University of Hong Kong, Hong Kong SAR, China

S1. Overview of the adapted cognitive-behavioural therapy sessions of older adults in the JoyAge project

Low-intensity (6 sessions, 2 hours each)	High-intensity (8 sessions, 2 hours each)
**1. Introduction to CBT and the group, mood check**<br>Ice-break, build relationships, and establish group rules; Introduce CBT goals, facilitate goal identification, practise mood-checks<br>*Homework: Mood check (encourage ≥3 times during the week)*	**1. Introduction to CBT and the group, mood check**<br>Ice-break, build relationships, and establish group rules; Introduce CBT goals, facilitate goal identification, practise mood-checks<br>*Homework: Mood check (encourage ≥3 times during the week)*
**2. How much do you know about emotions?**<br>Learn about common emotional reactions and their functions; Enhance awareness of bodily reactions amid stress and negative mood; Practise progressive muscle relaxation and breathing<br>*Homework: Mood check, stress reduction activities at least once and progressive muscle relaxation and breathing at least once (behavioural activation)*	**2. How much do you know about emotions?**<br>Learn about common emotional reactions and their functions; Enhance awareness of bodily reactions amid stress and negative mood; Practise progressive muscle relaxation and breathing<br>*Homework: Mood check, stress reduction activities at least once and progressive muscle relaxation and breathing at least once (behavioural activation)*
**3. Stress management**<br>Identify common causes and sources of stress, responses; Introduce stress management methods; Main components of CBT (cognition, emotion, behaviour, bodily reactions); Learn about symptoms of depression and their risk factors<br>*Homework: Mood check, stress reduction activities at least once (behavioural activation)*	**3. Stress management**<br>Identify common causes and sources of stress, responses; Introduce stress management methods; Main components of CBT (cognition, emotion, behaviour, bodily reactions)<br>*Homework: Mood check, stress reduction activities at least once (behavioural activation)*
**4. From "thoughts" to "emotions"**<br>Relationships between events and activities, thoughts, and emotions; Common thinking "traps", their impact on emotions, and facilitate awareness; Practise gratitude and positive thinking style<br>*Homework: Mood check, identify ≥1 negative/positive event and its relationship with behaviours, physiological reactions, thoughts, and mood, daily gratitude diary*	**4. From "thoughts" to "emotions" (1)**<br>Relationships between events and activities, thoughts, and emotions; Common thinking "traps", their impact on emotions, and facilitate awareness<br>*Homework: Mood check, identify ≥1 negative/positive event and its relationship with behaviours, physiological reactions, thoughts, and mood*
**5. New mindset, new lifestyle**<br>Identify own thinking traps, explore the role of negative cognitions, and personal coping methods; Practise questioning negative thoughts, build helpful thoughts (cognitive restructuring), and behaviours to improve mood (behavioural activation)<br>*Homework: Mood check, identify own coping strategies*	**5. From "thoughts" to "emotions" (2)**<br>Learn to be aware of one's own thinking "traps"; Learn about symptoms of depression and their risk factors; Set own activities for relieving negative emotions; Learning to engage in behaviours to modify mood<br>*Homework: Mood check, tracking of mood-enhancing behaviours and recording their mood*
**6. Start afresh**<br>Review CBT content, sharing of individual changes; Build personal tools to prevent depressive symptoms and design individual goals	**6. Gratitude**<br>Be aware of the importance and relevance of gratitude; Develop the capability to develop and express gratitude; Aware of the relevance of gratitude in positive thoughts and mood<br>*Homework: Mood check, identify ≥1 negative/positive event and its relationship with behaviours, physiological reactions, thoughts, and mood, daily gratitude diary*
**–**	**7. New mindset, new lifestyle**<br>Identify own thinking traps, explore the role of negative cognitions, and personal coping methods; Practise questioning negative thoughts, build helpful thoughts (cognitive restructuring), and behaviours to improve mood (behavioural activation)<br>*Homework: Mood check, identify own coping strategies*
**–**	**8. Start afresh**<br>Review CBT content, and sharing of individual changes; Build personal tools to prevent depressive symptoms and design individual goals
